# A Review on the Properties and Applications of WO_3_ Nanostructure-Based Optical and Electronic Devices

**DOI:** 10.3390/nano11082136

**Published:** 2021-08-22

**Authors:** Yu Yao, Dandan Sang, Liangrui Zou, Qinglin Wang, Cailong Liu

**Affiliations:** Shandong Key Laboratory of Optical Communication Science and Technology, School of Physics Science and Information Technology, Liaocheng University, Liaocheng 252000, China; lcuyaoyu0814@163.com (Y.Y.); zouliangruilcu@163.com (L.Z.)

**Keywords:** tungsten oxide, nanostructure-based, optical and electronic devices

## Abstract

Tungsten oxide (WO_3_) is a wide band gap semiconductor with unintentionally n-doping performance, excellent conductivity, and high electron hall mobility, which is considered as a candidate material for application in optoelectronics. Several reviews on WO_3_ and its derivatives for various applications dealing with electrochemical, photoelectrochemical, hybrid photocatalysts, electrochemical energy storage, and gas sensors have appeared recently. Moreover, the nanostructured transition metal oxides have attracted considerable attention in the past decade because of their unique chemical, photochromic, and physical properties leading to numerous other potential applications. Owing to their distinctive photoluminescence (PL), electrochromic and electrical properties, WO_3_ nanostructure-based optical and electronic devices application have attracted a wide range of research interests. This review mainly focuses on the up-to-date progress in different advanced strategies from fundamental analysis to improve WO_3_ optoelectric, electrochromic, and photochromic properties in the development of tungsten oxide-based advanced devices for optical and electronic applications including photodetectors, light-emitting diodes (LED), PL properties, electrical properties, and optical information storage. This review on the prior findings of WO_3_-related optical and electrical devices, as well as concluding remarks and forecasts will help researchers to advance the field of optoelectric applications of nanostructured transition metal oxides.

## 1. Introduction

The transition metal oxide tungsten oxide (WO_3_), an oxygen-deficient n-type wide band gap semiconductor material with an electronic bandgap of ~2.6–3.0 eV, has received a lot of attention [1,2,3,4]. WO_3_ structures include cubic, triclinic, monoclinic, orthorhombic tetragonal, and hexagonal. Because of its high melting temperature, photo electrochromic, toughness, and mechanical properties, it is regarded as a potential candidate for optical and electrical applications [5,6]. Nanostructured WO_3_ has a high specific surface area and good surface permeability, making it ideal for a wide range of applications. WO_3_ nanostructures in various morphologies (for example, instant nanorods (NRs), nanosheet, 3D nanostructured papilio paris, and thin films (TFs)) have been fabricated for a variety of applications, including gas sensors [7], efficient water splitting [8], photoelectrocatalytic activity [9], memory devices [10], photodetectors [11,12], and high temperature diodes [13,14]. At present, nanostructured WO_3_ are deposited on various substrates to fabricate optical and electrical devices, such as TiO_2_ [15], NiO [16], ZnO nanowires (NWs) [17], diamond [14], Fe_2_WO_6_ [8], and BiVO_4_ [18]. In the last few years, several review reports have been published based on photo catalysts [19,20], electrochromic devices [21,22], gas sensors [23,24], and oxygen-deficient WO_3_ [25]. However, so far there is no review focusing specifically on nanostructured WO_3_ optical and electrical devices.

As a result, in this review, the authors present a comprehensive overview of past developments in optical properties, such as photodetectors, light-emitting diodes (LED), photoluminescence (PL) and electrical properties, and optical information storage, as reported by various research groups, which make WO_3_ appealing for a variety of applications. Additionally, we offer some closing remarks as well as a forecast of the future advances in the subject. The research presented here should serve as a solid starting point for developing new nanostructured WO_3_ structures for emerging and future optical and electrical applications.

## 2. Photodetector

An ideal photodetector will display a low dark current to minimize the interference noise and high responsiveness to maximize the optical signal. The performance of photodetectors usually depends on the bandgap of the semiconductors and metal oxides. WO_3_ is a typical wide bandgap semiconductor with a large exciton binding energy of 0.15 eV, a high optical absorption coefficient ≥10^4^ cm^−1^, and a phonon-limited electron mobility of ~12 cm^2^ V^−1^ s^−1^. The WO_3_ band gap resonates with incident ultraviolet (UV) light energy, which can generate excess electrons that contribute to the photocurrent, thereby improving the characteristics of the photodetector [26]. These physical properties show that WO_3_ semiconductors have great potential for the fabrication of high-performance visible light and UV detectors with reasonable performance [27].

### 2.1. UV Photodetector

#### 2.1.1. Nanostructured WO_3_ Photodetector

**Figure 1:**
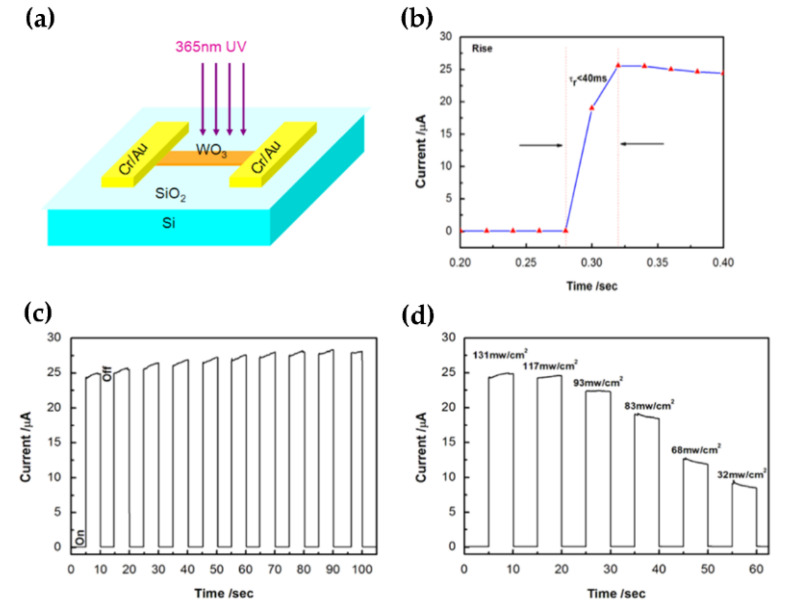
(**a**) Schematic of the device operation. (**b**) The photocurrent responses with time under the illumination of 365 nm. (**c**) The time-resolved photocurrent of the photodetector in response to light on/off at an irradiance of 131 mW/cm^2^ with 365 nm light. (**d**) The photocurrent–time curve with the change of light intensity. Reproduced or adapted from [31].

**Figure 2:**
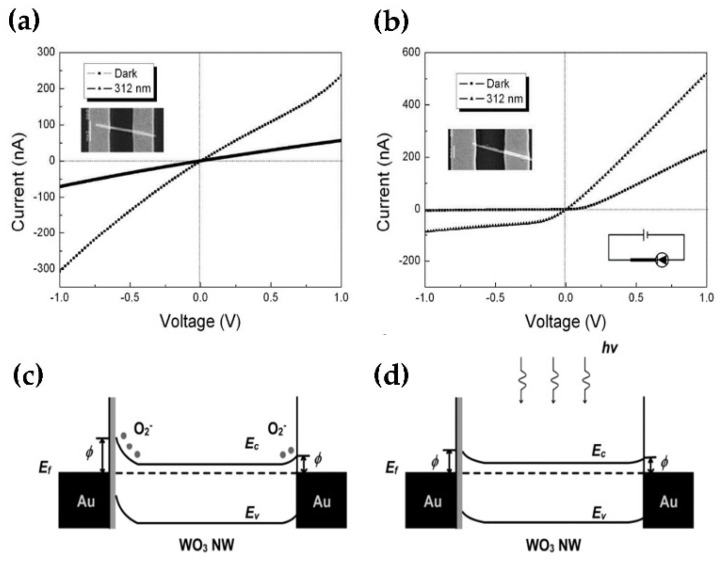
(**a**) A single WO_3_ nanodevice symmetrical device both in the dark and under 312 nm UV illumination, and the upper left inset is an SEM image of WO_3_ nanowire device. (**b**) *I-V* characteristics of the nonsymmetrical contact device, upper left inset is an SEM image of WO_3_ nanowire device and lower right inset shows the schematic structure of the device. (**c**) Band diagram of the Schottky barrier of the nonsymmetrical device in dark state. (**d**) Under UV illumination, the change of the barrier height. Reproduced or adapted from [32].

In recent years, there have been a few reports on the WO_3_ photodetector with a single nanostructure since its response time is slow and it has a low current on/off ratio [28,29,30]. In comparison to the previously described WO_3_ NWs [29,30] and nanospheres [28], Liu et al. have developed a photodetector based on a few layers of WO_3_ nanosheets that has a faster light response, a greater on/off ratio, a higher external quantum efficiency, high sensitivity, outstanding stability, and reversibility. This distinctive performance of WO_3_ photodetectors lays the foundation for the fabrication of high-performance flexible and multifunctional photodetectors derived from layered semiconductor materials (Figure 1a–d) [31]. To investigate UV photoresponse, symmetrical and asymmetrical standard lithography were evaluated to differentiate the sensitivity of the Ohmic contact and Schottky contact devices to UV photodetector based on a single WO_3_ NWs. The linear *I-V* curves of the symmetrical device in dark and UV illumination conditions demonstrated that the device is Ohmic contact. For Schottky contact device, one end of a WO_3_ nanowire was completely covered by the gold electrode from Omhic contact, and only very small area was covered at the other end. The *I-V* of a single WO_3_ nanowire device shows a typical diode *I-V* curve, and the effective circuit diagram in the lower-right inset. When reverse bias, the current was completely cut-off owing to the Schottky contact (Figure 2a,b). The Schottky contact device has a faster response time than the Ohmic contact device. This is due to the oxygen adsorption and desorption in the heterojunction influence the Schottky contact device barrier height. The adsorbed oxygen molecules on the surface of WO_3_ nanowire can modify the density of defect states and enhances the injection barrier (Figure 2c). Under UV illumination, the generated holes can release the adsorbed oxygen ions, and reduce the height of the injection barrier (Figure 2d). The reduction of the barrier height causes more electrons to cross the barrier and then enhances the photocurrent [32].

**Figure 3:**
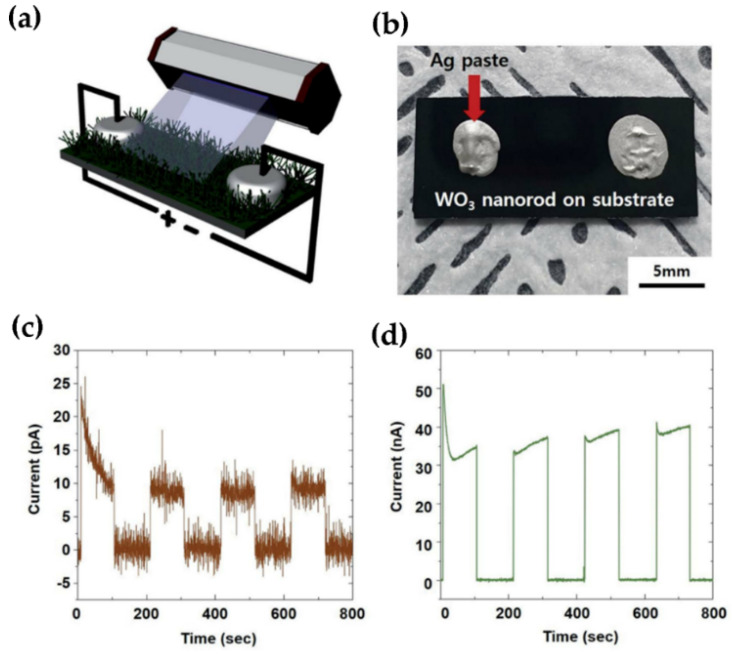
(**a**) The schematic illustration of performance measurement of self-crosslinked WO_3_ nanorods as a UV detector. (**b**) The photograph of the UV detector. The photocurrent response of UV-C ray (216 nm) irradiation of self-crosslinked WO_3_ (**c**) without Ag nanoparticles and (**d**) with Ag nanoparticles. Reproduced with permission from [33]. Copyright Royal Society of Chemistry.

In a recent study, Kim et al. heat-treated a large area of amorphous WO_3_ TFs to fabricate high quality, high-density WO_3_ NRs. It is used to prepare UV detectors, with a fast reaction speed (0.316 s), very sensitive to the actual UV 261 nm wavelength, and can effectively perform UV detection (Figure 3a–d) [33]. The carriers can be generated by transitions between bands caused by UV absorption. The generated carriers can also be transferred between each WO_3_ NRs. Finally, enough applied voltage can overcome the energy band barrier between the WO_3_ NRs and the electrodes.

#### 2.1.2. WO_3_ Thin Films for UV Photodetector

**Figure 4:**
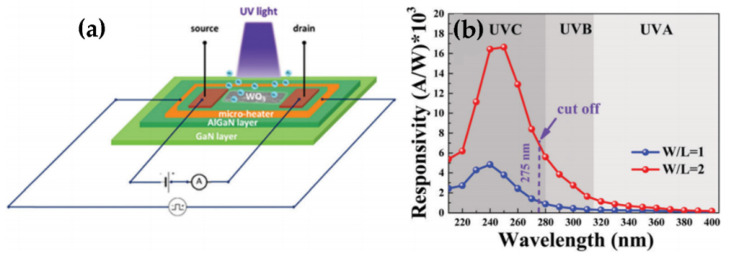
(**a**) Schematic illustration of the WO_3_/AlGaN/GaN heterostructure photodetector with an integrated micro-heater. (**b**) Measured spectral response of the WO_3_/AlGaN/GaN heterostructure photodetector. Reproduced or adapted from [11].

There are several reports on WO_3_ TFs based UV photodetectors. Reddy et al. studied the efficiency of UV photodetector characteristics by TiO_2_/WO_3_ bilayer TFs. The enhancement of oxygen vacancies in TiO_2_/WO_3_ bilayer plays an important role in photoresponse [34]. For the first time, high response and controllable recovery UV detector derived from a WO_3_ gate AlGaN/GaN heterojunction integrated microheater has been investigated (Figure 4a,b). The UV response rate of the device at 240 nm is 1.67 × 10^4^ A W^−1^, and the cut-off wavelength is 275 nm [11].

To improve the performance of the UV detector based on WO_3_ films, the sputtering parameters need to be optimized, mainly oxygen partial pressure and sputtering pressure. Yadav et al. described the effect of oxygen partial pressure on the performance of WO_3_ thin films UV detectors [35]. Sputtering technology is used to deposit WO_3_ film under different oxygen partial pressures to improve its response rate under low UV power density. The fabricated photodetector can respond to a small quantity of UV radiation. In addition, the effects of sputtering pressure on the morphology, crystallinity, and photodetector properties of WO_3_ films were also investigated [12]. WO_3_ film has high crystallinity, surface roughness, and customized grain size, which contribute to achieving high responsivity and external quantum efficiency by minimizing the overall impedance. The results show that the WO_3_ film deposited under a sputtering pressure of 10 m Torr has good stability and high photodetector performance.

**Figure 5:**
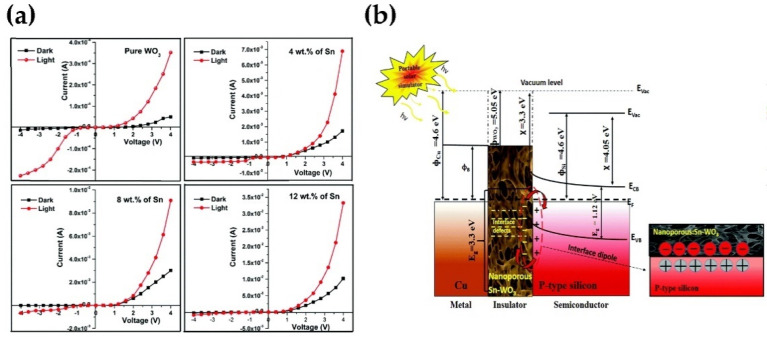
(**a**) *I-V* characteristics Cu/nanoporous: Sn–WO_3_/p-Si SBDs fabricated with different concentrations (**b**) Energy band diagram of the Cu/nanoporous:Sn–WO_3_/p-Si (MIS) type diode. Reproduced or adapted from [38].

Furthermore, the oxygen vacancies were found as doubly ionized donors and help with photodetection at the same time. In addition, Sn ions into the WO_3_ lattice can enhance the conductivity and reduce the resistivity by increasing the carrier concentration and oxygen vacancy. The prepared WO_3_ and Sn–WO_3_ precursor solutions of 3 mL were deposited separately on the cleaned p-Si substrates (1 cm × 1 cm) by the jet nebulizer spray pyrolysis technique with different concentrations of Sn (0, 4, 8, and 12 wt.%) the copper (Cu) metal contact was coated on the Sn–WO_3_/p-Si using dc sputtering (Model Name: HIND high vacuum-PS 2000) with 4 mm diameter and 500 nm thickness. The instrument details and deposition conditions of the metal contacts were mentioned in their previous work [36,37]. The Sn-WO_3_/p-Si diode showed a positive light response of high reverse saturation current under illumination [38]. As the concentration increases, the detection capability of the interface layer also increases. The diode measured under light conditions exhibits higher current values than under the dark conditions. This behavior outcome indicates that all the Cu/Sn–WO_3_/p-Si diodes are highly photo-conducting in nature. In particular, the diode fabricated with 12 wt.% of Sn shows higher current values (mA level) when compared to other diodes (Figure 5a). It is presumed that the incorporation of Sn atoms in the WO_3_ matrix has effectively improved the photocurrent of the Cu/Sn–WO_3_/p-Si diodes. The ideality factor (n) of the diodes was found to reduce steeply under dark conditions on increasing the Sn concentration from 0 to 12 wt.%. Compared to the dark conditions, the diodes measured under light conditions revealed lower n values. This is mainly due to the increase in the photo-generated charge carriers (e^−^–h^+^) along with the improved conversion efficiency of the semiconductor layer and absorption of interfacial layer (Figure 5b).

### 2.2. Visible Photodetectors

**Figure 6:**
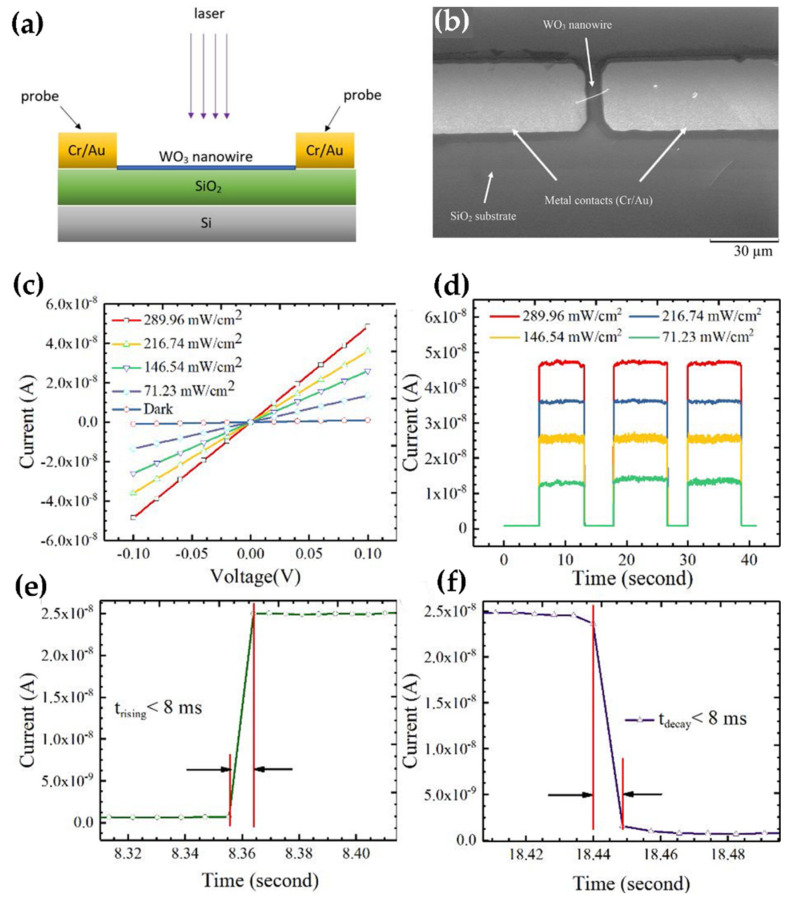
(**a**) Sketch of the WO_3_ nanowire device structure; (**b**) SEM image of the WO_3_ nanowire device. (**c**) Current-voltage curves and (**d**) photo-switching behavior of the WO_3_ nanowires photodetector under the illumination of a 404 nm laser with different laser intensities. (**e**,**f**) are the rising time and decay time of the WO_3_ nanowire photodetector. Reproduced or adapted from [27].

The nanostructures of WO_3_ and the UV detectors have been investigated, and device characteristics have been reported. However, there is not much research on the application of WO_3_ as visible light detectors. Wang et al. reported on the controlled synthesize of WO_3_ NWs and their application as visible photodetectors. The WO_3_ single NWs photodetector has shown outstanding device characteristics with a high responsivity of 19 A W^−1^ at 0.1 V, high detectivity of 1.06 × 10^11^ Jones, and a short response time of 8 ms under a 404 nm laser illumination (Figure 6a–f). Thus, the enormous possibility of WO_3_ NWs for manufacturing visible photodetectors has been established [27].

**Figure 7:**
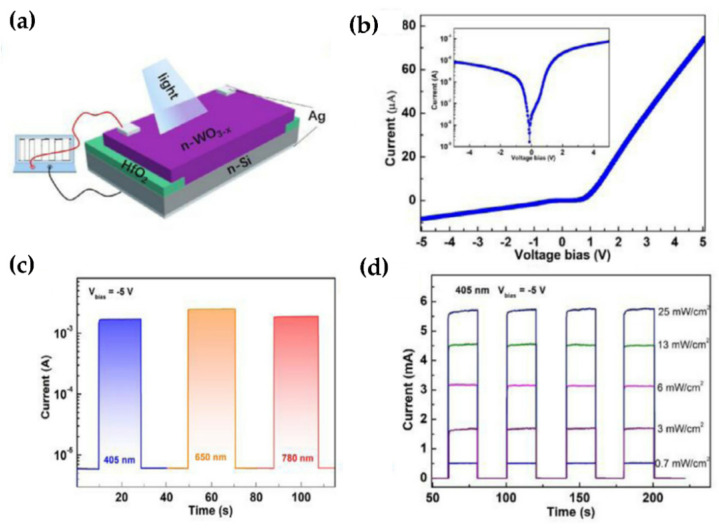
(**a**) The schematic illustration of the WO_3−x_/Si n-n homotype heterojunction. (**b**) The *I-V* curve of a typical WO_3−x_/Si n-n homotype heterojunction in dark at room temperature, inset shows the semi-logarithmic *I-V* curve. (**c**) The time response under various light signals at −5 V bias. (**d**) The time responses at −5 V bias under pulsed 405 nm light illumination with different light intensities. Reproduced or adapted from [39].

Sub-stoichiometric WO_3−x_/Si n-n homo-heterojunction with rectification properties has been fabricated by Zhang et al. (Figure 7a–d) [39]. The heterojunction shows remarkable photodetection performance involving a high specific detectivity of 3.96 × 10^11^ Jones, a large responsivity of 72.8 A/W, and fast response times of 5.8 μs/1.27 ms under 405 nm. The WO_3−x_/Si n-n homo-heterojunction has the potential for superior visible photodetector characteristics with the scope for optoelectronic applications.

## 3. Light-Emitting Diode

**Figure 8:**
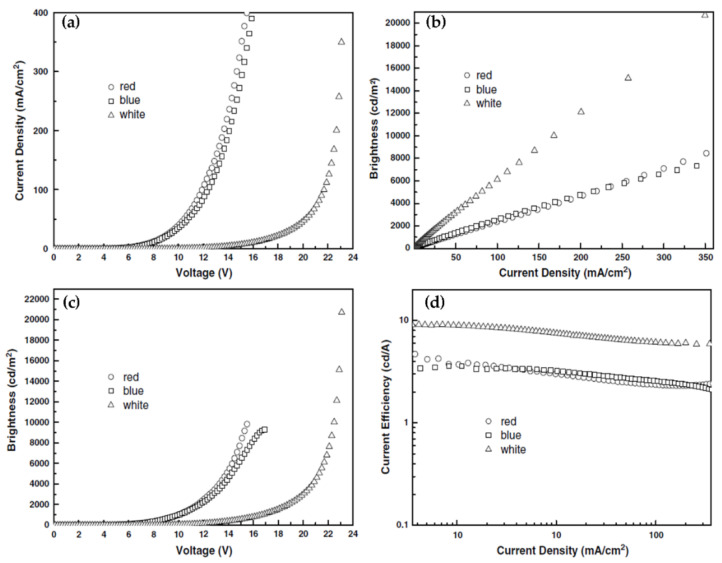
EL performance of tandem white OLED (triangles), the control blue device (squares), and the control red device (circles): (**a**) current–voltage, (**b**) brightness–current, (**c**) brightness–voltage, and (**d**) current efficiency–current. Reproduced or adapted from [41].

**Figure 9:**
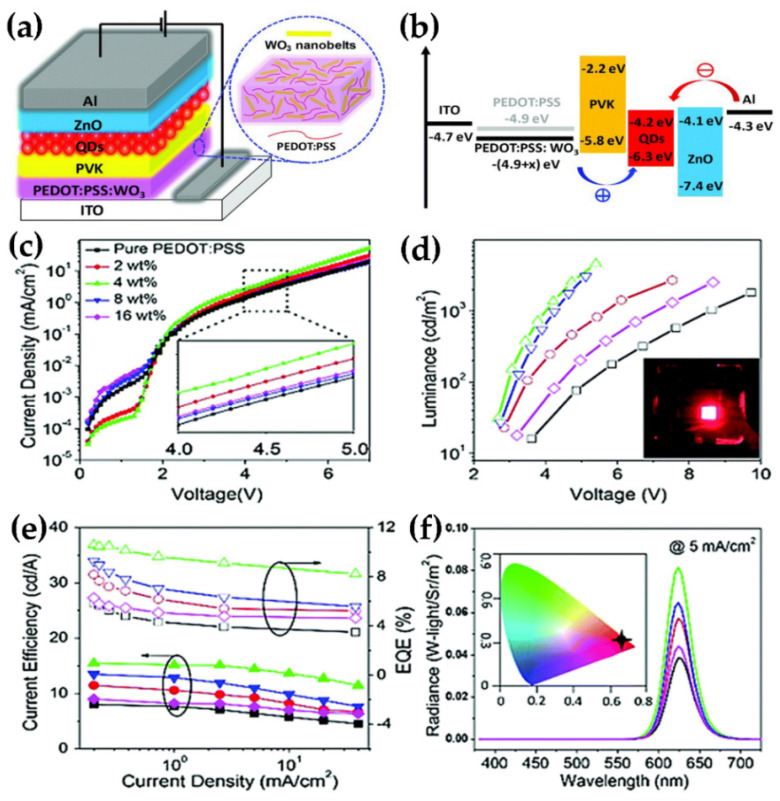
(**a**) Schematic device structure. (**b**) Energy level diagram of QLEDs device. (**c**–**f**) show the device performance of red QLEDs with different concentrations of WO_3_ (0–16.0 wt.%) doped PEDOT:PSS as the hole injection layer. (**c**) Current density–voltage characteristics. (**d**) Current density–luminance characteristics. Inset: the device driven at 5 mA cm^−2^. (**e**) Current efficiency–current density–EQE characteristics. (**f**) Electroluminescence (EL) spectra at 5 mA cm^−2^. Inset: Commission Internationale de l’Eclairage (CIE) coordinates of a typical red-QLED. Reproduced or adapted from [45].

WO_3_ has a low emission efficiency due to the presence of a highly linked metal ion polyhedron [40], which limits its use in light-emitting diodes (LEDs). Researchers have fabricated tandem organic light-emitting diodes (OLEDs) connected to WO_3_ intermediate connections in the early days to fully utilise the photoelectric capabilities of WO_3_ and make it more useful in LEDs [41,42,43,44]. Zhang et al. used an Al/WO_3_/Au structure as the interconnection layer of tandem white LED. Through the microcavity effect, this connecting layer makes white light emission more stable and eliminates the angular dependence of the spectrum generated by the microcavity effect. The design of this laminated structure provides an idea for the realization of higher-efficiency organic white LEDs. Performance comparison of a serial OLED with a single LED is shown in Figure 8. Compared with traditional LEDs, serial OLEDs display higher current efficiency, brightness, and longer operating life [41]. Wei et al. investigated a pure blue OLED with a blue fluorescence emitter, in which the charge producing layer was made up of transparent WO_3_ TFs and the electron transport layer was Li-doped. When compared to single emitting unit devices, series devices have significantly more power and longer service life [42]. Bao et al. studied the electronic structure and the energy level arrangement of WO_3_-based intermediate connectors. The authors have explained the significance of using WO_3_ as an interlayer—it can significantly change the energy level arrangement, make the interface dipole and energy level bend, and facilitate the injection of carriers into the appropriate molecular energy level of the adjacent electroluminescent unit [43]. Bin et al. have fabricated a tandem stack to enhance the electroluminescence property of white OLED. In the tandem laminate, 1,4,5,8,9,11-hexaazatriphenylene hexacarbonitrile (HAT-CN) is used as the organic charge generation layer and WO_3_ is used as the inorganic charge generation layer. In double-stack OLEDs, it is observed that WO_3_, as the charge generating layer, has the best performance with exceptional CIE color coordinates [44]. Researchers have observed that doping nanostructured WO_3_ into high molecular polymers (PEDOT:PSS, PANI:PSS) [45,46] as the hole injection layer in classic OLEDs, may be a good choice. Using a simple solid-state mechanochemical approach, Zhuo et al. have produced WO_3_ nanoribbons with a width of 10 nm and a length of 80 nm and doped them into PEDOT:PSS as a mixed hole injection layer for QDLEDs [45]. Figure 9a,b shows the device structure and its energy level diagram. Compared with the QDLED with PEDOT:PSS as the hole injection layer alone, the QDLED based on the mixed hole injection layer shows high external quantum efficiency(EQE) and stronger current efficiency, as shown in Figure 9c–f. Zhu et al. synthesized WO_3_ nanocrystals hybridized with conductive polymer (PANI:PSS) and used them in the hole injection layer of OLEDs. The hybrid system reduces the surface defects of WO_3_ nanocrystals and improves the interface contact ability. Compared with OLED containing only WO_3_ nanocrystals and traditional PEDOT: PSS-WO_3_ devices, this device exhibits higher capacitance and stronger luminous efficiency. PANI:PSS-WO_3_ composite material is expected to be a candidate material as the charge injection layer in new generation OLEDs [46].

## 4. Photoluminescence Properties

WO_3_ has been extensively explored in electrochromic, photochromic, and gas sensing materials, and other applications due to its exceptional physical and chemical features. However, being an indirect bandgap semiconductor, WO_3_ shows lower emission efficiency, which makes its light-emitting characteristics poor. Manfredi et al. investigated light emission in WO_3_ TFs at liquid nitrogen temperatures in the early years. The light emission ceases while the TFs are at room temperature, indicating that studying the PL of WO_3_ at room temperature is not acceptable [47]. Researchers have put in a lot of effort to study WO_3_’s light emission at ambient temperature. For example, Niederberger et al. [48,49,50]. achieved room temperature blue emission of WO_3_ nanoparticles in ethanol solution. Later, Khold et al. have shown that the morphology, particle size, and quantum confinement play a critical role in the luminescence at room temperature [51], which indicates how to improve the luminescence of WO_3_ at room temperature. On this basis, Feng et al. prepared crystalline WO_3_ TFs with different nanostructures by thermal evaporation of tungsten wires and observed strong PL at room temperature [52]. Wang et al. synthesized WO_3_ nanosheets on a large-scale using tungsten powder as raw materials and demonstrated its blue emission at room temperature [53]. Park et al. synthesized needle-like nanostructures of WO_3_ by thermal evaporation in the temperature range of 590 °C to 750 °C and explained the influence of substrate temperature and the morphology of WO_3_ nanostructures on luminescence [54].

**Figure 10:**
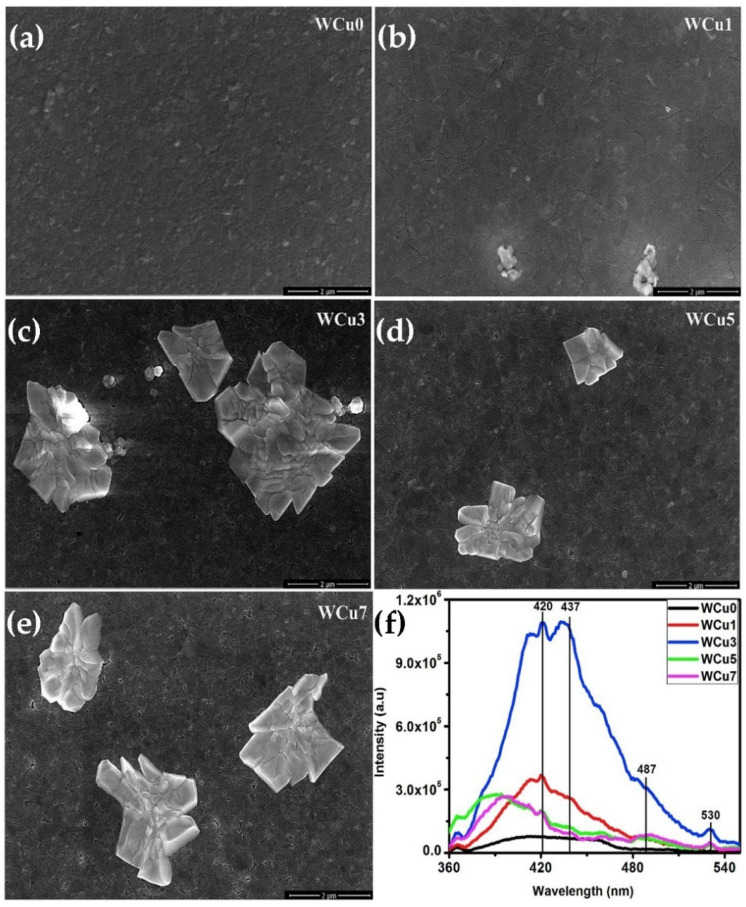
(**a**–**e**) FESEM images and (**f**) Photoluminescence emission spectra of the undoped and Cu doped WO_3_ thin films. Reproduced or adapted from [60].

**Figure 11:**
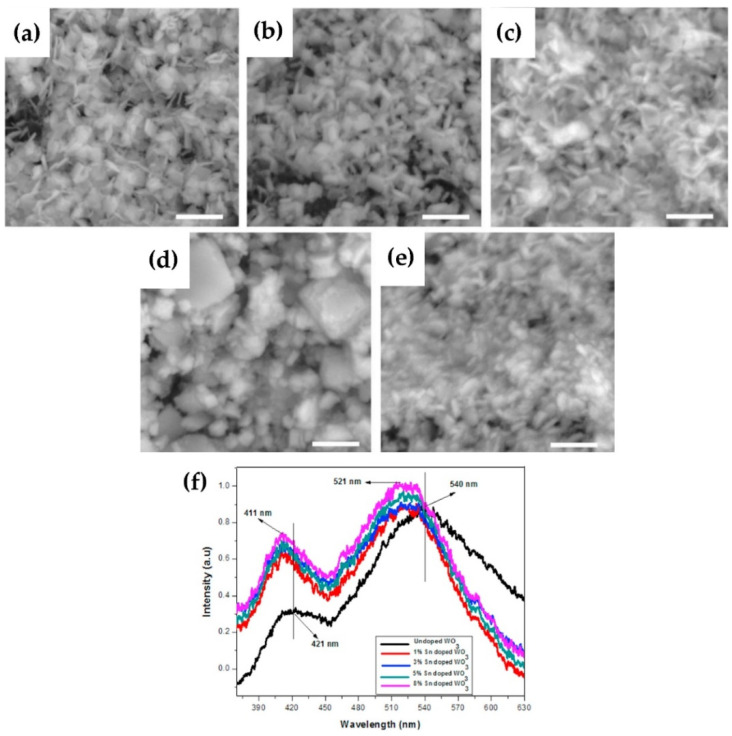
SEM images of (**a**) undoped (**b**) 1% (**c**) 3% (**d**) 5% and (**e**) 8% Sn doped WO_3_ nanostructures. Scale bar is 500 nm. (**f**) PL spectra of undoped and Sn doped WO_3_ nanostructures. Reproduced or adapted from [59].

Except for the methods mentioned above, doping with rare-earth ions (Eu^3+^, Tb^3+^) [40,55,56] or metal ions (Li, Sn, Cu) [57,58,59,60] in WO_3_ is considered as another effective method to improve luminescence performance at room temperature. Luo et al. prepared Eu^3+^ doped WO_3_ TFs by hydrothermal method. They found that with the increasing of Eu ion content, the morphology of WO_3_ changed significantly. Moreover, Eu^3+^ doping significantly improved the transparency and optical contrast of WO_3_ [55]. Ruan et al. prepared WO_3_:Eu^3+^ inverse opal photonic crystals and studied their luminescence characteristics. The crystal generated red PL at 615 nm and showed a better red purity [56]. Kavitha and his colleagues used RF magnetron sputtering to prepare high-quality and efficient luminescent Tb^3+^-doped WO_3_ TFs, which showed strong green, blue, and red emission under UV excitation [40]. In addition, they also used the same technique to prepare Cu-doped WO_3_ TFs. The plasmon resonance behavior of Cu nanoparticles on the TFs has been studied, and it has been observed that they can considerably improve the quantum efficiency of various photonic devices and that Cu doping causes the TFs to emit intense blue light (Figure 10) [60]. Kovendhan et al. reported the effect of Li doping (1–5 wt.%) with different contents into the WO_3_ TFs. They proposed that the structure and optical characteristics of WO_3_ TFs changed with the increase of lithium, and observed blue PL emission that was difficult to detect at room temperature. The bandgap values of WO_3_ TFs doped with Li (1–5 wt.%) shifted blue, and the blue emission increased dramatically, as compared to pure WO_3_ TFs [57]. Mukherjee et al. investigated the preparation of Sn-doped WO_3_ TFs by chemical spray pyrolysis. They found that the peak intensity of near-band edge emission in doped TFs was enhanced relative to that in undoped TFs, and the spectral intensity was enhanced with the increase of Sn content [58]. Sn-doped WO_3_ nanosheets were produced using a simple precipitation process by Mehmood et al. and their photoelectric characteristics were examined. SEM images of WO_3_ nanosheets with different Sn doping levels (0–8 wt.%) are shown in Figure 11a–e. Similar to the results reported by Mukherjee et al. [58], they found that Sn doping significantly enhanced the PL intensity of WO_3_ nanosheets and resulted in a shift in the near-band edge emission transition (Figure 11f) [59].

## 5. Electrical Properties

WO_3_, despite its unique properties of high thermal stability, superior charge transport, tunable electrical properties, and high electron mobility, is not commonly used in the electrical device sector [61,62,63,64]. The electronic devices are used in a variety of environments, including humid, dry, and high-temperature environments, as well as in the dark or under light irradiation. In recent years, researchers are looking at the electrical features of WO_3_ nanostructures and TFs, including electrical transport properties, electrical conductivity, field emission mechanism, and resistance switching behavior.

### 5.1. Nanostructured WO_3_ Electrical Properties

Throughout the last decade, nanoscale electronic and optoelectronic devices involving nanometer-sized inorganic systems have been shown to have comprehensive electrical properties that are sensitive to form and size [65,66,67]. As such, researchers have paid close attention to the electrical characteristics of WO_3_ nanoparticles.

WO_3_ is one of the typically unintentionally doped n-type characteristic semiconductors [68]. WO_3_ often presents sub-stoichiometric oxide (WO_3−x_) due to the presence of several oxygen deficiencies, such as WO_2.9_, WO_2.83_, WO_2.8_, and WO_2.72_. That is to say, the lattice of WO_3−x_ could sustain a considerable amount of oxygen vacancy and contain a number of W^5+^. Consequently, change of oxygen vacancies in WO_3−x_ could effectively tune the density of electron, and then have considerable conductivity [69,70,71]. WO_3_ that strictly satisfy the stoichiometric ratio should be an insulator, and non-stoichiometric WO_3−x_ exhibits n-type semiconductor behavior. A slight change in oxygen content can also greatly change the conductivity of WO_3_, so its electrical properties vary with its oxygen content and can be divided into exhibiting metal and semiconductor behavior. Extrinsic n-doping is therefore not required for WO_3_ to exhibit significant conductivity. Due to the greater bandgap of quasi-two-dimensional (Q2D) WO_3_, Q2D WO_3_ nanoflakes have more potential electrical applications. Zhuiykov et al. investigated the electrical characteristics of orthorhombic-WO_3_ nanoflakes with thicknesses ranging from 7 to 9 nm [72]. Sun et al. prepared high-quality WO_3_/CoWO_4_ core-shell p-n junction NWs by hydrothermal method. A physical model of Ag ions spreading along core-shell NWs to form conductive wires was proposed to interpret the bipolar resistance switching behavior. The new WO_3_/CoWO_4_ core-shell p-n junction NWs are suitable for the next generation of nonvolatile memory [73].

#### 5.1.1. Oxygen Vacancy Effect

The separate oxygen vacancies effect on WO_3_ electrical characteristics has been a source of debate [74,75]. The electrical characteristics of tungsten oxides with various oxygen vacancy levels are important to investigate. The thermal evaporation produced nano/microrods with the same morphology that show varying oxygen vacancy content as the WO_3−x_ level increased [76]. The devices composed of WO_3−x_ crystals have shown a negative to positive humidity resistance response. The device’s conductivity was boosted with more oxygen vacancies as a result of a more photogenerated carrier transit and effective separation. The ability to manufacture a range of optoelectronic devices and humidity sensors has been demonstrated by the WO_3−x_ crystal. The current-voltage characteristics related to the Au/WO_3_ NW/Au devices show that with the increase of bias voltage and oxygen vacancy concentration, the conduction mechanism of the devices changes from volume-limited (space charge-limited) to electrode-limited (Schott Base launch) as have been reported by Yang et al. By adjusting the concentration of oxygen vacancies and even the scanning range of the bias voltage, the resistance switching behavior of WO_3_ NWs can be adjusted [77].

#### 5.1.2. Electrical Transport Behavior

**Figure 12:**
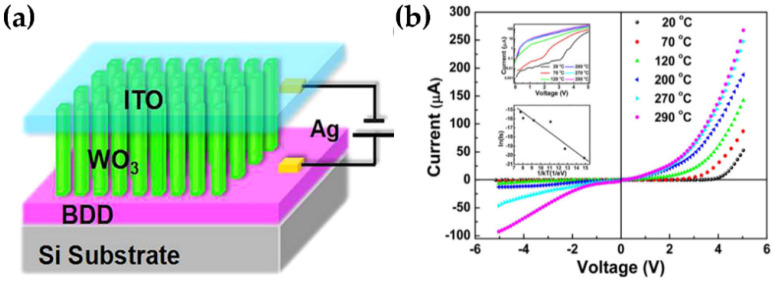
(**a**) Schematic diagram of the n-WO_3_NRs/p-BDD heterojunction device. (**b**) I-V plots of the n-WO_3_ NRs/p-BDD heterojunction working at varying temperatures from 20 °C to 290 °C. The top inset is the plot of log (Current) vs. voltage, and the bottom inset is the plot of ln(Is) vs. 1/kBT to obtain the activation energy. Reproduced from [14], with the permission of AIP Publishing.

In recent years, a few applications have focused on the electrical transport characteristics of the WO_3_ nanostructures. Li et al. studied the high-temperature electrical transport characteristics of hydrothermally produced n-WO_3_ NRs/p-diamond heterojunctions. Within the temperature range of room temperature to 290 °C, the p-n heterojunction displayed excellent thermal stability and rectification properties (Figure 12a,b). With increasing temperature, the turn-on voltages decreased and the rectification ratio increased. This research broadens the design and application possibilities for heterojunctions based on BDD, particularly at high temperatures, high power, and in a variety of hostile environments [14].

Khan et al. studied the dielectric and electrical transport mechanism of multilayer flower-like WO_3_ microstructure by impedance spectroscopy. The equivalent circuit model is used to explain the impedance plane. The electrical transport properties of WO_3_ were studied in detail. The low dielectric loss at 1MHz makes WO_3_ a potential material for high-frequency applications. Reproduced or adapted from [78].

#### 5.1.3. Field Emission Properties

WO_3_ NWs show high conductivity owing to abundant oxygen vacancies, which is contributed to the progress of its FE performance. Previous studies have also shown that WO_3_ NWs have excellent FE performance as a cold cathode potential.

**Figure 13:**
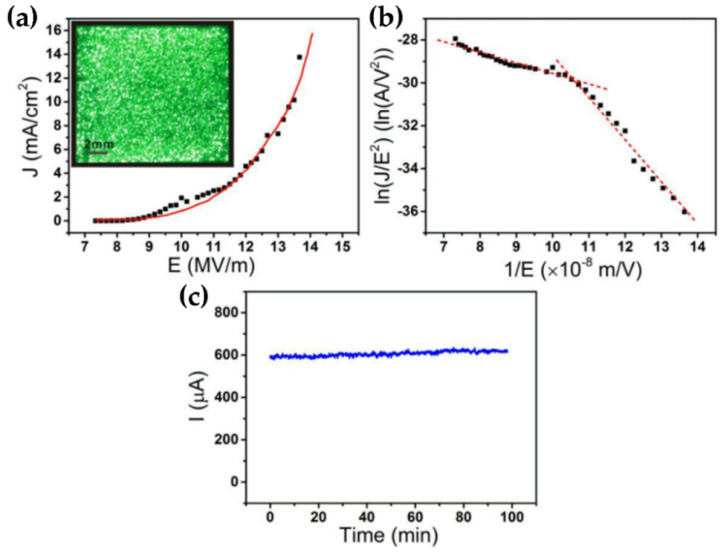
(**a**) FE *J–E* curve of the large-area patterned defect-rich WO_3_ NWs film. The emission image (**b**) corresponding F–N plot and (**c**) emission current stability of the patterned WO_3_ NWs film. Reprinted (adapted) with permission from [80]. Copyright {2021} American Chemical Society.

A non-catastrophic breakdown phenomenon was found during the FE process of single defect WO_3−x_ NWs, which can extend the life of the electron source of the NWs. The main reasons for this phenomenon are the defect-related electrical transport-induced breakdown mechanism and the Nottingham effect-induced cooling impact. The detection provided a practical method for designing a single NW point source with a long lifespan, which was critical to the advancement of high-performance semiconductor NW point sources [79]. Uniform large area and micropatterned WO_3_ NWs were fabricated and their FE properties were investigated by Lin et al. A high FE current up to 3.5 mA was obtained in a defect-rich WO_3_ sample with an effective area of 0.25 cm^2^ (Figure 13a–c). The high emission current was incited by the high defect density in the WO_3_ NWs [80].

A field emission study of well-aligned uniform WO_3_ nanoconifer was carried out at ~1 × 10^−8^ mbar pressure. The turn-on and threshold field values were achieved to be 2.43 and 3.08 V µm^−1^, respectively. The findings of the WO_3_ nanoconifer FE characteristics investigation suggest that it could be a good candidate for multifunctional applications like cold cathodes in nanoelectronic devices.

### 5.2. WO_3_ Thin Film Electrical Properties

**Figure 14:**
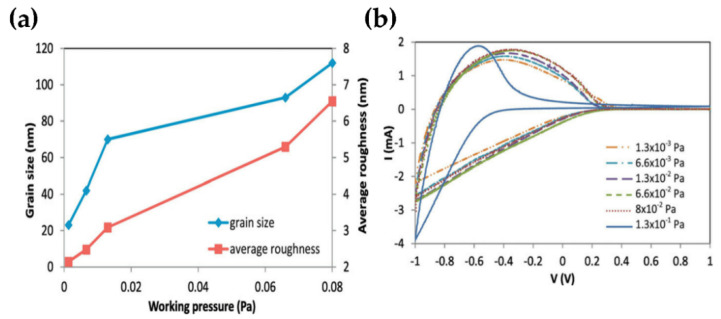
(**a**) Variation of grain size and average film roughness with the working pressure. (**b**) Cyclic voltammetry for WO_3_ films deposited at different working pressures. Reproduced or adapted from [83].

WO_3_ TFs have gained importance in recent years, both in terms of basic research [81] and in terms of their application potential as energy-saving smart windows and batteries [82]. Although many studies have focused on using nanostructured WO_3_ TFs to increase film optical efficiency, nothing has been done to optimize their electrical properties. However, their mixed conductivity (ionic and electronic) is particularly significant. Samad et al. have studied the relationship between the nanostructure and electrical properties of amorphous WO_3_ TFs. The ionic conductivity and lithium chemical diffusion coefficient are explored to increase with the supplement of the grain size while the conductivity is proposed to diminish with increasing grain size (Figure 14) [83].

**Figure 15:**
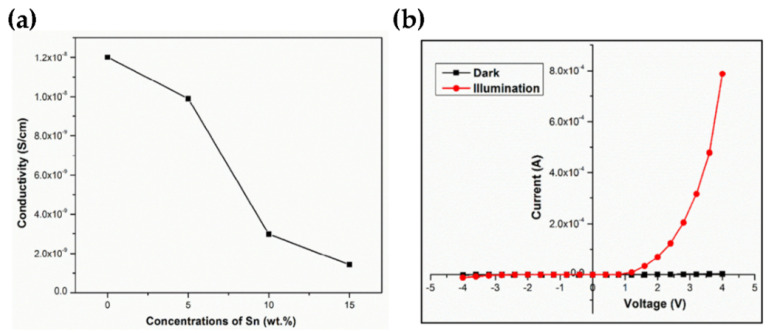
(**a**) The average conductivity of 0, 5, 10, and 15 wt.% of Sn_0.26_WO_3_ thin films (**b**) I-V characteristics of 15 wt.% of the n-Sn_0.26_WO_3_/p-Si diode in darkness and under light illumination. Reproduced or adapted from [84].

Shanmugasundaram et al. used spray atomizer pyrolysis technology to prepare Sn_0.26_WO_3_ TFs with Sn (0, 5, 10, 15 wt.%) and n-Sn_0.26_WO_3_/p-Si heterojunction diode 15 wt.% doping concentration at 500 °C substrate temperature. The *J-V* curve indicates that the 15 wt.% Sn doped WO_3_ TFs demonstrate a minimum conductivity of 1.429 × 10^−9^ S/cm. From the diode characterization under illumination and in dark, the acquired ideality factor of n values are 4.89 and 5.03 for 15 wt.% of n-Sn_0.26_ WO_3_/p-Si heterojunction diode (Figure 15) [84].

**Figure 16:**
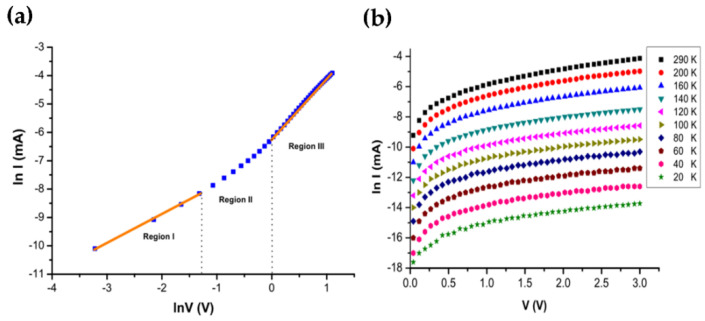
(**a**) The ln*I*-ln*V* plot of p-SnS/n-WO_3_:Sb heterojunction at 300 K. (**b**) ln*I-V* plots of p-SnS/n-WO_3_:Sb heterojunction at various temperatures. Reproduced or adapted from [13].

The electrical transport mechanism of p-SnS/n-WO_3_:Sb TFs heterojunction in the temperature range of 20–300 K was investigated. At low forward bias voltage (<0.25 V), the diode shows Ohmic conduction. At the middle voltage range (0.25 < V < 1.0 V), the carrier transport mechanism followed the thermionic emission. When the forward bias voltage is above 1.0 V, the current transport is space charge limited current (SCLC) according to the exponential trap contribution in the WO_3_:Sb bandgap. (Figure 16a) The temperature dependence of the ideality factor n and saturation current can be interpreted by tunneling enhanced recombination model emerging at the interface the heterojunction with E_00_ and E_a_ values about 99.5 meV and 1.565 eV, respectively (Figure 16) [13].

**Figure 17:**
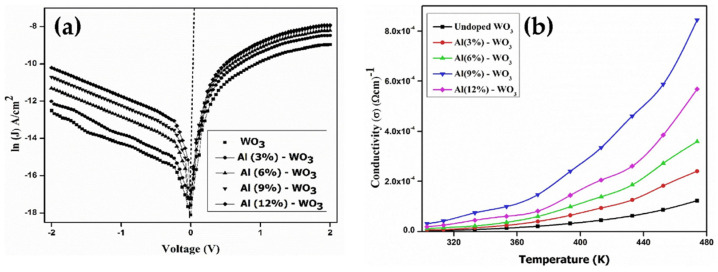
(**a**) ln(*J*)-*V* characteristics of the WO_3_/p-Si diodes for different dopant concentrations of Al. (**b**) Electrical conductivity of the WO_3_ thin films for different dopant concentrations of Al as a function of temperature. Reproduced or adapted from [86].

Pure and Al:WO_3_/p-Si heterojunction diode was fabricated by the sol-gel spin coating method. The electrical conductivity of the WO_3_ TFs increases with Al (0–9 wt.%) dopant concentration in the temperature range of 303–473 K (Figure 17). The *J-V* characteristic of Al:WO_3_/p-Si diode shows the decreasing barrier heights Φ_B_ at the lower temperature, The decreasing barrier height with low temperature is easily understood considering that the current becomes gradually controlled by electrons that can cross the lower barrier patches, which reduces apparent barrier height. Supposing a Gaussian spatial distribution for Φ_B_, with mean Φ_Bm_ and the standard deviation σ_B_, the temperature dependence of the measured barrier height Φ_B_ at zero applied bias is expected to follow the relation [85]: $\Phi_{B}=\Phi_{\mathrm{Bm}}-\frac{{q\sigma }_{B}^{2}}{2\mathrm{KT}}$. The results show that 9wt.% Al:WO_3_/p-Si diodes have better performance than other diodes [86].

## 6. Memory Application

Since traditional silicon-based storage technology will soon reach its limit, this brings a serious challenge to the scaling of the device size. To overcome this limitation, researchers have been trying to find new materials to develop new information storage technologies. In recent years, to solve the scaling limitations of traditional flash memory, research on non-volatile memory has become a top priority. At present, the research on non-volatile memory has become matured. The common non-volatile memory includes magnetic random access memory (MRAM), ferroelectric random access memory (FRAM), resistance random access memory (RRAM), and phase change random access memory (PCM). Compared with other memories, resistance random access memory (RRAM) is easily manufactured, a simple device structure, low operating voltage, high durability, high-density storage, long retention time, fast switching speed, and excellent performance. It has the advantages of scalability and compatibility with traditional complementary metal-oxide-semiconductor (CMOS) technology [87,88]. To exhibit high performance, the size of the memory device should be kept small, because the large devices exhibit higher noise levels and poor reproducibility. This is due to uncontrollable deviations and defects within the larger devices [89]. Therefore, building nano-level devices has become a necessity to solve this problem.

Because of its outstanding performance in three-dimensional stacking, compatibility with CMOS process [90,91,92], good durability [93], non-volatile rectification characteristics [94] with excellent electrochromic and photochromic properties, and other factors, nanostructured tungsten oxide (NWs, NRs, nanosheets, etc.) has been widely used in RRAM devices in recent years. Among these advantages, the electrochromic properties of WO_3_ are one of the criteria that determines the storage performance of RRAM. The degree of crystallinity of tungsten oxide determines its electrochromic performance. Compared with crystal-oriented tungsten oxide, amorphous tungsten oxide shows better electrochromic performance. However, amorphous tungsten oxide exhibits poor durability in acid electrolyte solutions [95]. To solve the above problems, researchers have studied wet chemical preparation methods to construct tungsten oxide composite systems, such as electrodeposition [96,97] and sol-gel methods [98,99]. Shim et al. prepared polycrystalline WO_3_ NWs on ITO substrates by electrospinning. Compared with traditional WO_3_ nano-films, they found that the prepared WO_3_ NWs showed faster charge transfer, better optical response, and better pigmentation efficiency, and the memory effect after pigmentation also significantly improved [100]. Pang et al. synthesized WO_3_ composite TFs modified with Ag nanoparticles by a combination of vacuum deposition and electrodeposition methods. Compared with pure WO_3_ TFs, the films show stronger electroactivity and electrochromic properties [101].

**Figure 18:**
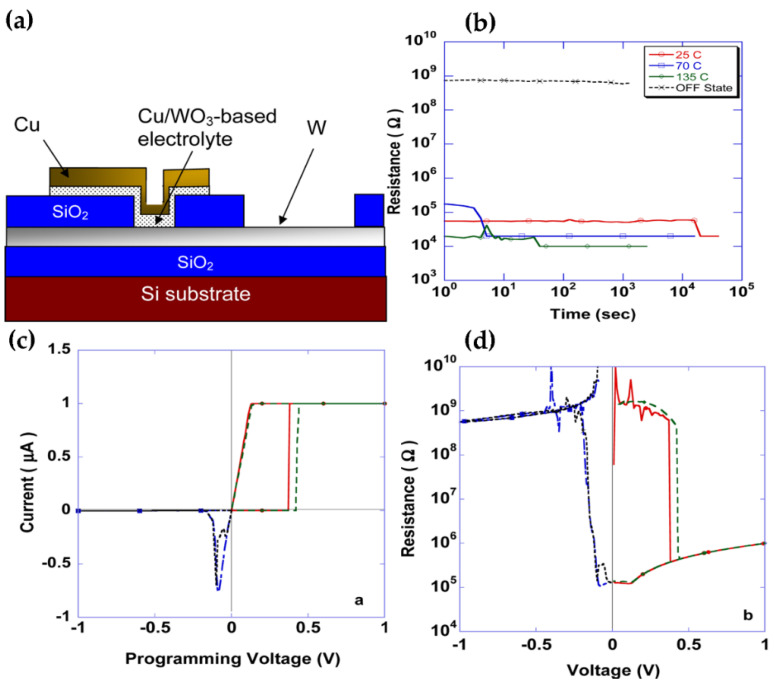
Diagram of cross-section and electrical behavior of Cu-doped WO_3_ devices. (**a**) Diagram of a cross-section of device. The region of the active device is the W-electrolyte-Cu layer superimposed together, and its area is defined by the diameter of the through-hole in the SiO_2_ dielectric. (**b**) Data retention behavior of Cu-doped WO_3_ devices at 10 μA and 1 V. (**c**) I-V diagram of the device at 1 μA. (**d**) R-V plot of the device. Reproduced or adapted from [90].

Kozicki et al. demonstrated that low-power RRAM devices doped with copper WO_3_ are achievable. This type of device exhibits a high turn-off resistance and can be switched to an on-resistance state under a low voltage state. This state is independent of the device geometry but is strongly controlled by the current. If a small reverse bias is applied, they can be restored to a high-impedance state. Schematic diagram of the device structure and its resistance switching characteristic is shown in Figure 18. Unfortunately, the manufacture of such devices requires the diffusion of copper light into WO_3_, which will be difficult to apply in semiconductor processing [90]. On this basis, Li and co-workers fabricated RRAM devices using Cu/WO_3_/Pt structures. This device exhibits better resistive switching characteristics, such as better durability, lower power consumption, and better retention. In addition, they also explained the physical mechanism of the multi-level storage characteristics in Cu/WO_3_/Pt storage devices. The origin of these multi-level storage characteristics can be explained as that when a higher current is applied to the device, the radial growth of conductive filaments and the formation of more conductive filaments together, lead to this characteristic [92,102]. Inspired by this phenomenon, Kozicki et al., Li et al., and Sun et al. synthesized high-quality WO_3_/CoWO_4_ core-shell NWs using the hydrothermal method. They studied the bipolar resistance switching behavior of Ag/[WO_3_/COWO_4_]/Ag devices using Ag as an electrode. They found that the device maintained excellent stability for 100 cycles and had an on/off ratio of up to 333 at room temperature [73]. Similarly, Chakrabarti et al. designed a new RRAM device with W/WO_3_/WO_x_/W structure and observed the effects of post-annealing of metal on the behavior of shapeless resistance switch in this structure, especially the F-N tunneling effect after LRS and reset. Furthermore, for all nonlinear current-voltage switching characteristics, the authors have used simulations to account for SCLC conduction in low voltage field, F-N tunneling in high voltage field, and oxygen vacancy carbon fiber with a diameter of ~34 nm. This work will contribute to comprehend the switching mechanism of other similar RRAM structures and selector-free nanoscale crossover structures [103].

**Figure 19:**
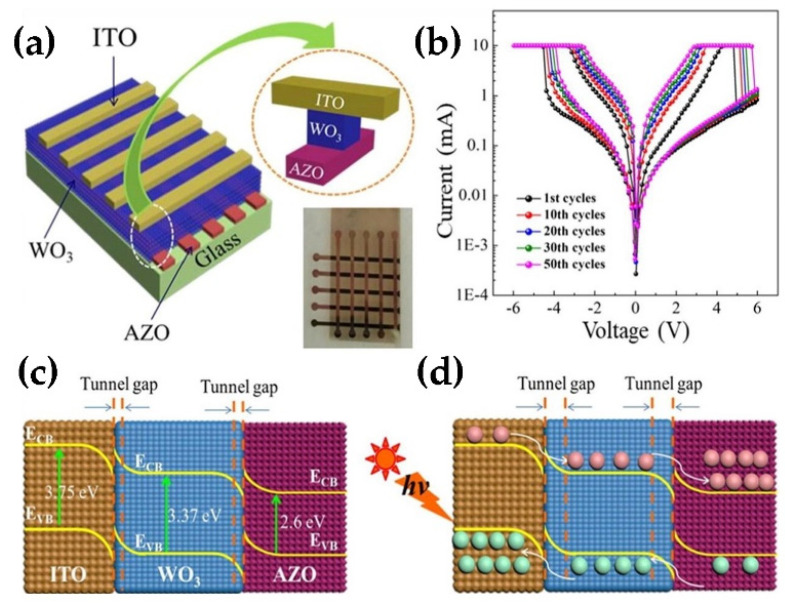
(**a**) Structure diagram of ITO/WO_3_/AZO device. (**b**) The sample was annealed at 600 °C and then irradiated for 30 min, followed by 50 consecutive cycles of volt-ampere characteristic curve in logarithmic form. (**c**) Energy band structure of ITO/WO_3_/AZO. (**d**) Schematic diagram of carrier migration and interfacial tunnel gap formation under light irradiation. Reproduced or adapted from [104].

**Figure 20:**
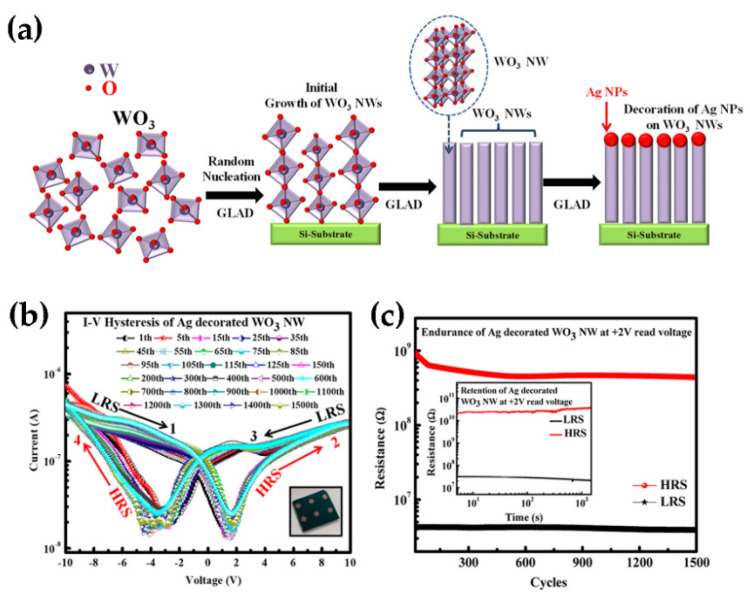
(**a**) Schematic illustration of the growth processes of Ag-decorated WO_3_ NWs. (**b**) Semi-logarithmic scale volt-ampere characteristic curve of Ag-decorated WO_3_ NWs device at room temperature. The inset shows the image of Ag-decorated WO_3_ NWs devices. (**c**) Switching durability of Ag-decorated WO_3_ NWs at +2 V read voltage. The inset shows the data retention characteristics of Ag-decorated WO_3_ NWs devices. Reprinted (adapted) with permission from [105]. Copyright {2021} American Chemical Society.

Recently, Sun et al. have fabricated ITO/WO_3_/AZO resistance switching devices by a magnetron sputtering and observed that the devices have significantly enhanced resistance-switching memory behavior. The schematic diagram of the ITO/WO_3_/AZO device and I-V characteristic curve are shown in Figure 19. Subsequently, they proposed a physical model of photogenerated carriers tunneling in the Schottky barrier layer driven by electrical pulses to fully explain this phenomenon. The deployment of non-volatile RRAM devices in future development will be guided by this physical model [104]. Singh and his colleagues synthesized Ag-decorated WO_3_ NWs by the glancing angle deposition (GLAD) technique. The growth process of Ag-decorated WO_3_ NWs is shown in Figure 20a. Figure 20b shows the I-V characteristic curves of the device, exhibiting a large storage window of ~ 12.02 V at ±10 V and a low interface density trap of ~5.74 × 10^10^ eV^−1^ cm^−2^ at 1 MHz. It also exhibits an on/off switching time lasting up to 1500 cycles (Figure 20c) and a stable retention time of up to 10^3^ s on/off resistance ratio (~245) [105]. Moreover, the same technology was used to synthesize WO_3_ NWs based capacitive memory on Si substrates. Through the measurement and analysis of the device performance, the authors found that the memory exhibited a stable retention time (10^3^) and a good endurance cycle of up to 100 [106]. These works provide interesting ideas for the design and application of next-generation non-volatile memory.

## 7. Conclusions and Future Outlook

Recent research has significantly increased our knowledge of the features and uses of WO_3_-related nanostructures in optical and electrical devices, such as photodetectors, LEDs, PL properties, electrical properties, and optical information storage devices. Although WO_3_ based devices exhibit good performance in the fields of various photoelectrical applications, it is also desirable to find new fabrication routes and the types of electrodes for these devices to improve the optical and electrical properties in the future. Modifying the electrode configuration of the device, controlling the variety of morphologies of WO_3_ nanostructures, and optimizing the preparation process could be the effective strategy for various futuristic photoelectrical applications. In order to better understand the physical transport mechanism of the devices, it is critical to use more relevant semiconductor theory and computational models for carrier transport research toward the development of WO_3_ devices.

Up to present, the WO_3_ nanostructure related tunnel diode with negative differential resistance (NDR) investigation is scarcely researched, NDR is a non-linear carrier transport phenomenon, whereby the electrical current decreases with increasing bias voltage. N-WO_3_ semiconductor exerts degenerative features through heavy n-type doping and possibly displays a NDR phenomenon when combined with p-degenerative semiconductor. The NDR effect of WO_3_ will make an important contribution to the implementation of logic switches, oscillators, inverters, resistive switching memory, and radiation reliable device applications in the field of flexible electronics semiconductors.

Over the past decades, the development of the optoelectronic applications based on the WO_3_ nanostructures that can operate in harsh environments (high temperature or strong radiation environments) is still challenging. It is also necessary to put further efforts to investigate WO_3_ related optical and electrical devices under extreme conditions such as high temperature, high pressure, and harsh environments. Diamond is an excellent semiconductor material for manufacturing high-performance electronic devices that are used in high temperatures and a strong radiation environment. Therefore, it is expected to fabricate WO_3_/diamond heterojunction device for providing the possibility of photodetectors, LEDs, PL properties, and optical information storage devices application at higher temperatures.

## Data Availability

Data available in a publicly accessible repository.
